# Synthesis and Evaluation of Anticancer Activity of New 4-Acyloxy Derivatives of Robustic Acid

**DOI:** 10.3390/ijms20215336

**Published:** 2019-10-26

**Authors:** Rui Chen, Lini Huo, Yogini Jaiswal, Jianhua Wei, Dianpeng Li, Jing Zhong, Leonard Williams, Xing Xia, Yan Liang

**Affiliations:** 1 School of Chemistry and Chemical Engineering, Guangxi University, Nanning 530004, China; 58251323@163.com; 2 Faculty of Chinese Medicine Science, Guangxi University of Chinese Medicine, Nanning 530222, China; 3 College of Pharmacy, Guangxi University of Chinese Medicine, Nanning 530222, China; huolini@126.com (L.H.); weijianhua@163.com (J.W.); zhongjing1212@163.com (J.Z.); xiaxing66@163.com (X.X.); 4 Center for Excellence in Post-Harvest Technologies, North Carolina A&T State University, The North Carolina Research Campus, 500 Laureate Way, Kannapolis 28081, NC, USA; yoginijaiswal@gmail.com (Y.J.); llw@ncat.edu (L.W.); 5 Guangxi Institute of Botany, Chinese Academy of Sciences, Guilin 541006, China; 6 College of Pharmacy, Guangxi Medical University, Nanning 530021, China; vincyliang@163.com

**Keywords:** robustic acid, *Dalbergia benthamii* Prain, DNA topoisomerase I, anticancer

## Abstract

In the present study, a series of 4-acyloxy robustic acid derivatives were synthesized and characterized for evaluation of their anti-cancer activity. The structures of these derivatives were elucidated by mass spectra (MS) nuclear magnetic resonance spectra (NMR). The single-crystal X-ray diffraction structure of one of these compounds was obtained, for further validation of the target compound structures. The anticancer activities of the target products were evaluated against human leukemic cells HL-60, human non-small cell lung carcinoma cells A-549, human hepatic carcinoma cells SMMC-7721, human hepatocellular carcinoma cells HepG2, and human cervical carcinoma cells Hela. Three compounds among them exhibited potent in-vitro cytotoxicity and excellent DNA topoisomerase I inhibitory activity, even at 0.1 mM concentrations. The most noteworthy observation was the minor toxicity of two of these compounds to normal cells, with an activity similar to the positive control in cancerous cells. A Surflex-Dock docking study was performed to investigate the topoisomerase I activity of all compounds. Of all the other compounds, the most sensitive compound was selected for further investigation of its effect on apoptosis induction and cell cycle regulation in HL-60 cells. Our results suggest that the anticancer effects of these compounds can be attributed to their pharmacological effects on topoisomerase I, cell apoptosis, and cell cycle. These findings suggest that robustic acid derivatives could be used as potential antitumor drugs.

## 1. Introduction

**Figure 1:**
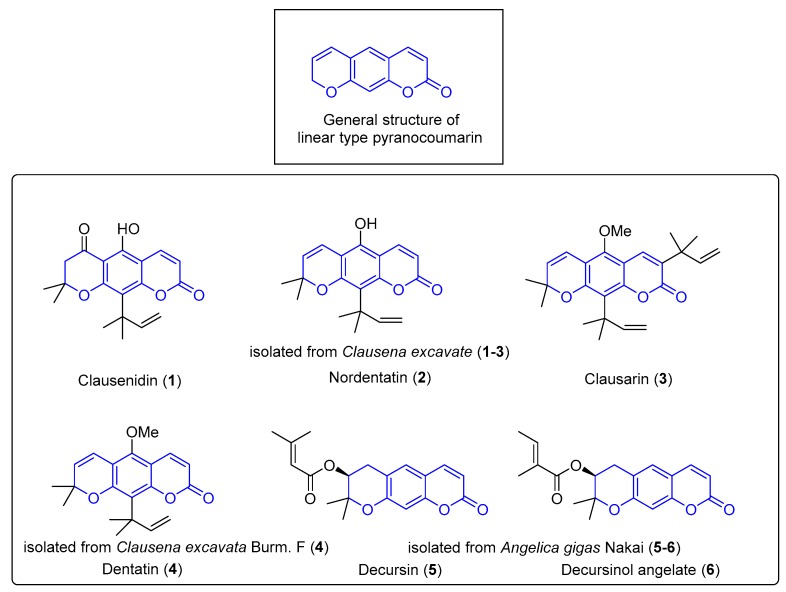
Chemical structures of some linear-type pyranocoumarins.

Coumarin compounds are widely distributed in nature, especially in higher plants [1]. They are proven to possess a wide range of biological activities, with minimal side effects [2]. In particular, pyranocoumarins (including linear type and angular type) are an interesting class of fused coumarins. They are important as photoactive drugs with important pharmacological effects that include anti-inflammatory [3], anti-HIV-1 [4], anti-hepatitis B virus [5], anti-cancer [6], and antinociceptive properties [7]. As the major constituents from *Clausena* or *Angelica* genus plants [8,9,10,11], some linear-type pyranocoumarins (shown in Figure 1) with anti-cancer property have become compounds of growing research interest. Examples of such compounds are, clausenidin (**1**), nordentatin (**2**), clausarin (**3**), dentatin (**4**), decursin (**5**), decursinol angelate (**6**), etc. Compounds 1–3 mentioned above are reported to exhibit potent cytotoxicity against human non-small cell lung carcinoma cells (A549), human breast adeno carcinoma cells (MCF-7), human nasopharyngeal carcinoma cells (KB), and multidrug-resistant nasopharyngeal carcinoma cells (KB-VIN) [5]. Compound 4 had significant antitumor activity on MCF-7, human hepatocellular carcinoma cells (HepG2), and human prostate carcinoma cells (PC-3 and LNCaP) through causing the induction of apoptosis [12,13,14]. Compounds **5** and **6** are more cytotoxic to leukemia cancer cells [15,16]. However, detailed information on the cytotoxic mechanism and structure-activity relationships of these linear-type pyranocoumarins is still limited in the literature. Considering the wide spectrum of biological activities of these compounds, the synthesis of new linear pyranocoumarins has drawn significant attention in recent years. 

**Figure 2:**
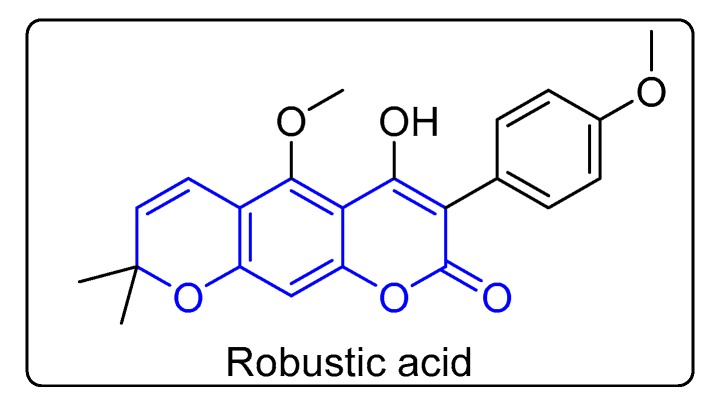
Structure of robustic acid (RA).

Recently, our group isolated and identified a new linear pyranocoumarin was from *Dalbergia benthamii* Prain: robustic acid (RA; Figure 2). RA constitutes to about 3.5% *w/w* of its dry matter and was considered to be one of the major bioactive phytoconstituents of *D. benthamii* [17]. *D. benthamii* is used commonly in traditional medicine for the treatment of traumatic injury, as a pain reliever and anti-inflammatory agent, and for regulating menstrual cycle [18]. To further explore and continue our earlier investigation on linear pyranocoumarins, the synthesis of a new series of 4-acyloxy compounds of robustic acid derivatives was carried out. Preliminary cytotoxic activities and related mechanisms such as DNA topoisomerase I (Topo I), cell cycle, and apoptosis were also investigated in this study.

## 2. Results and Discussion

### 2.1. Chemistry 

**Scheme 1:**
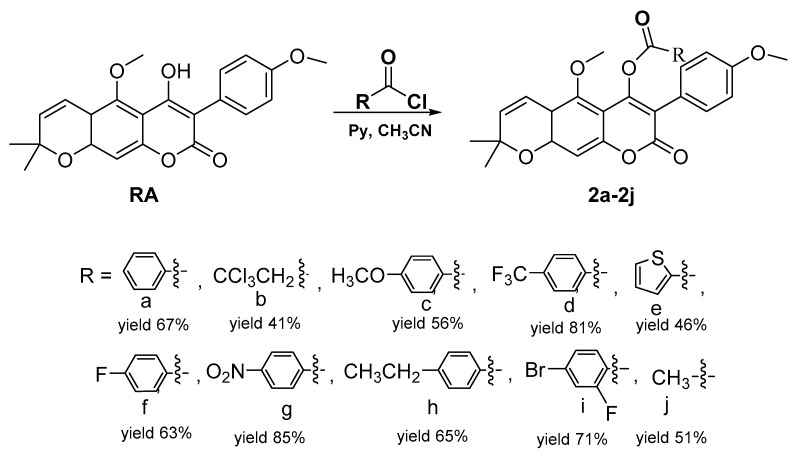
The synthesis of 4-acyloxy robustic acid derivatives (**2a**–**2j**).

**Figure 3:**
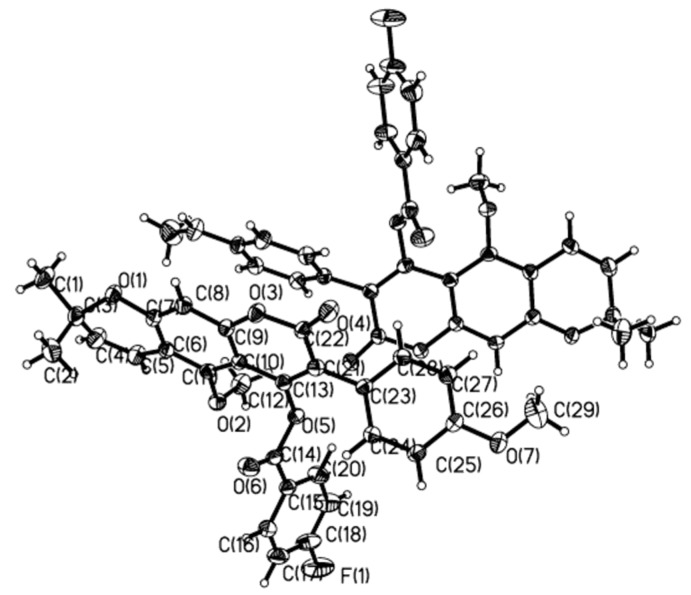
Crystal structure of compound **2f**.

**Figure 4:**
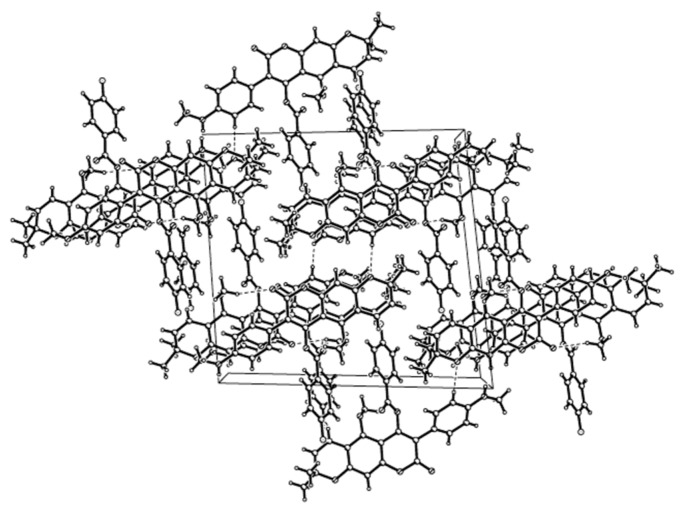
View of the packing compound **2f** in the cell.

The scheme used for the synthesis of 4-acyloxy robustic acid derivatives (**2a**–**2j**) is illustrated in Scheme 1. The starting material RA was isolated from *D. benthamii* in our previous study [17]. The target compounds **2a**–**2j** were synthesized by nucleophilic substitution from RA and derivatives of acyl chloride. We found that the yields of aromatic acyl chloride compounds were higher than the fatty acyl chloride compounds, which ranged from 41–85%. With the attachment of the withdrawing group on aromatic ring, the reaction time was shortened to less than 1 h and the yield was greatly improved. The structures of compounds were elucidated by, MS, ^1^H NMR, and ^13^C NMR. All spectral data were in accordance with the assumed structures, and are listed in the procedure. The ESI-MS indicated that the molecular weights were in accordance with the calculated values. In ^1^H NMR spectra, the single peaks of two methoxyl groups of the original nucleus appeared at δ 3.79 and δ 3.56, and six hydrogen atom signals of two methyl groups were observed at δ 1.47–1.59 ppm. Two pyran ring hydrogen signals were in the ranges δ 6.02–6.58 and δ 5.50–8.74, respectively. Further verification of the structures of the target products was carried out, and a single crystal for **2f**(CCDC 1944893, these data can be obtained free of charge via http://www.ccdc.cam.ac.uk/conts/retrieving.html or the Cambridge Crystallographic Data Centre, 12 Union Road, Cambridge CB2 1EZ, UK; fax: (+44) 1223-336-033; or e-mail: deposit@ccdc.cam.ac.uk.) was obtained in ethanol solution. The molecular structure of **2f** is shown in Figure 3, and the packing diagram of **2f** is depicted in Figure 4. The formation of a twin crystal was observed in the crystal structure of **2f**. Most bond lengths in the system fell in the range of single and double bonds and the C–C bond distances in all rings ranged between 1.316(5) and 1.498(6) Å. These values are almost equal to the values of typical bonds of aromatic structure. The crystal packing of the compound is stabilized by π–π stacking.

### 2.2. Biological Study

#### 2.2.1. Anti-Proliferative Activity Screening

All the synthesized compounds were evaluated for their cytotoxicity effects in five human cancer cell lines and two normal cells. The cell lines included: leukemia cells HL-60, human non-small cell lung carcinoma cells A549, human hepatic carcinoma cells SMMC-7721, hepatocellular carcinoma cells HepG2, cervical carcinoma cells Hela, human normal liver cells LO2, and human normal lung epithelial cells BEAS-2B. The obtained IC_50_ values of the synthesized compounds, compared to RA and the reference drug (cisplatin and camptothecin (CPT)), are shown in Table 1. Interestingly, the activities of most of the target products were superior to the parent nucleus. Compounds **2d**, **2g**, and **2i** exhibited promising biological activity in almost all cell lines. Of all the cell lines selected, they were more sensitive to HL-60, with IC_50_ values of 21.04 ± 0.43, 16.63 ± 0.12, and 16.38 ± 0.27 μM, respectively. The obtained IC_50_ values were close to the positive control cisplatin (14.23 ± 0.56) and CPT (12.25 ± 1.06). In addition, **2d** and **2g** exhibited moderate activity in Hela cells, with IC_50_ values of 37.03 ± 0.97 and 27.45 ± 0.38 μM, respectively. Their potential antitumor activity might be related to the attracting group on benzene ring of 4-aroyl. It should be noted that most target compounds could significantly inhibit the proliferation of selected cancerous cells with no damage to normal cells (LO2 and BEAS-2B (IC_50_ > 100 μM)). However, compound **2g** has detrimental effects on LO2 cells, as it exhibited an IC_50_ value of 20.79 ± 0.97 μM. All compounds were less toxic on non-cancerous cells than positive control. This indicates that these compounds (especially **2d** and **2i**) have highly selective activity against cancerous cells and are non-toxic to normal human cell lines. They can thus be suggested as potential candidates for development into anti-tumor drugs with high efficiency and low toxicity.

#### 2.2.2. Evaluation of DNA Topoisomerase I (Topo I) Inhibitory Activity and Molecular Docking Study

**Figure 5:**
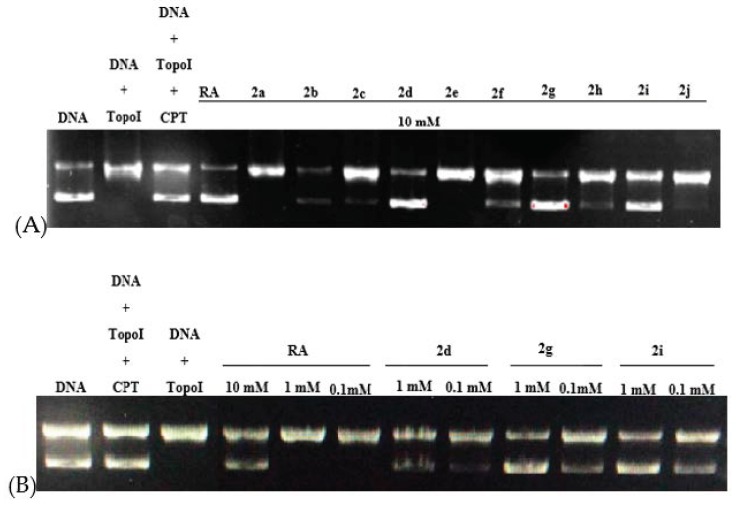
DNA topoisomerase I (Topo I) inhibitory activity of (**A**) CPT, RA, and all target compounds (**2a–2j**) at 10 mM; (**B**) Compounds **2d**, **2g**, and **2i** in different concentrations using CPT (0.1 mM) as a positive control.

DNA Topo I is an important enzyme in all living organisms, and it participates in many cellular metabolic processes, such as replication, transcription, recombination, and repair [19]. DNA Topo I has been established as a molecular target of anticancer drugs. A combined study of synthesis and docking for the anti-cancer activity of novel coumarins concluded that coumarin derivatives are potent cytotoxic agents. Docking studies further suggest that all coumarins possess higher binding affinities with DNA Topo I [20]. Currently, discovering novel non-camptothecin inhibitors for DNA Topo I has become an important area of research. Thus, a DNA Topo I inhibitory activity assay was also carried out using the supercoiled DNA unwinding method in this study [21]. Figure 5A shows the conversion of supercoiled plasmid DNA Topo I in the presence of compounds RA and **2a** to **2j** with camptothecin (CPT) as a positive control. Compounds **2d, 2g**, and **2i** showed significant inhibitory activities at 10 mM concentration. Compounds **2b**, **2c**, **2f**, and **2h** showed moderate inhibitory activities with obscure strips in electrophoresis. Compounds **2a**, **2e**, and **2j** did not show any considerable Topo I inhibitory activity. Therefore, compounds **2d**, **2g**, and **2i** were selected for further quantitative investigation (depicted in Figure 5B). It was found that the inhibition weakened gradually as the concentration of RA reduced. At critical concentrations lower than 10 mM, no inhibitory activity was found. In agarose gel electrophoresis, DNA fragmentation was evident with treatment of **2d**, **2g**, and **2i**, even at low concentrations of 1 mM or 0.1 mM. Note that **2g** and **2i** exhibited Topo I inhibitory activities higher than that of compound **2d**. This correlates well with the cytotoxicity to HL-60, A-549, and SMMC-7721 cells. Thus, we infer that the anti-proliferative activity of 4-acyloxy derivatives of RA to these three cells may be related to Topo I.

**Figure 6:**
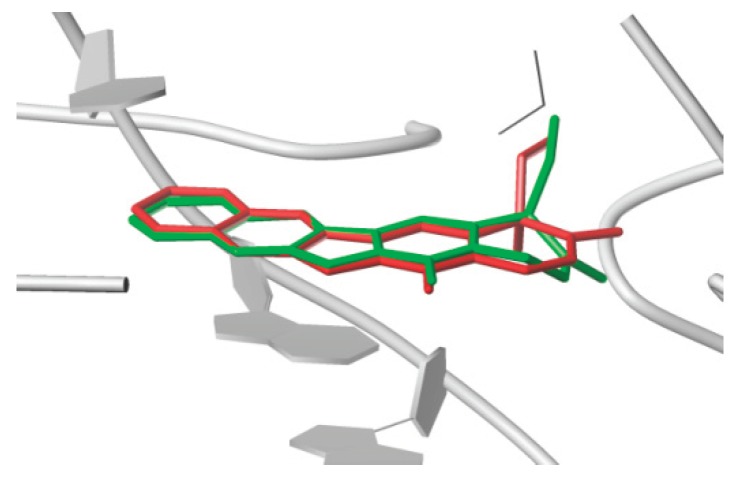
Result of validation for Topo I inhibitor (CPT) inside the enzyme active site (derived from PDB ID 1T8I).

To understand the mechanism of Topo I inhibitory activity of the compounds synthesized, molecular docking studies were carried using the Surflex-Dock algorithm of Sybyl-X 2.0 (Tripos Inc., St. Louis, MO, USA). The crystal structure of DNA Topo I in complex with CTP was downloaded from the Protein Data Bank (PDB code: 1T8I). To validate the molecular docking approach, the crystallographic pose of CTP (derived from complex) and the top-ranked docking pose obtained in this study were compared. The two binding poses of CTP had almost perfect superposition, with an RMSD (root-mean-square deviation) value of 0.4438 Å (Figure 6). The obtained RMSD value is far below the well-established tolerance level of 2.0 Å, thus validating the adopted docking methodology [22,23].

**Figure 7:**
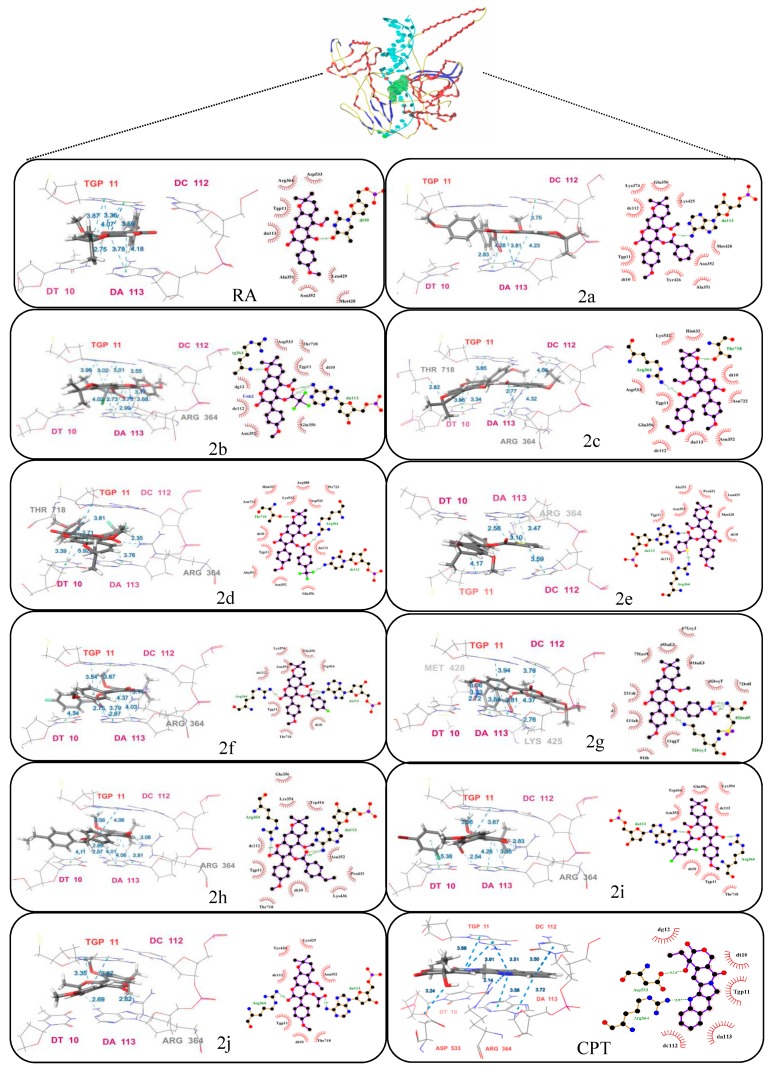
The best pose of the binding mode of compounds with DNA Topo I complex (PDB:1T8I).

After running Surflex-Dock, the binding affinities of protein–ligand complexes were obtained and expressed as total score. All 10 inhibitors were docked into the active site of Topo I, as shown in Figure 7. The predicted binding affinities (total score) of all the compounds containing hydrophobic residue, hydrogen bond, and π–π stacking are listed in Table 2. The results indicate that all synthetic compounds except **2b** had better combination with Topo I than the RA mother nucleus. Compound 2g exhibited the highest total score value of 10.02, which was close to the positive control (CPT). The most important residues on the **2g**–Topo I complex (Figure 4h) were ILE427, TGP11, DT10, DA113, ARG364, DC112, PRO357, GLU356, LYS374, GLU418, and TYR426. As depicted, the oxygen atom of acyl group of compound **2g** made a hydrogen bonding interaction with the hydrogen of the -NH_2_ group of LYS425 (O–H-LYS425, 2.76 Å). The oxygen atoms in the –NO_2_ group made three binding interactions with hydrogens of the -NH_2_ group of MET428 (O–H-MET428, 2.72, 3.06, and 3.33Å). Compounds **2d** and **2i** also exhibited good binding affinities, with total scores of 8.76 and 8.89. All these compounds exhibited remarkable Topo I inhibitory activity even at low concentration, which is nearly in agreement with our observations of in vitro studies. However, the results of the docking assay did not exactly correlate with the Topo I inhibitory activity. The binding affinity of all compounds were decreasing in sequence of **2g**, **2h**, **2i**, **2c**, **2d**, **2e**, **2f**, **2a**, **2j**, **2b**. However, the inhibitory activities of **2h** and **2c** were not as good as the prediction data. This may be related to other combination modes or other pharmacological mechanisms.

#### 2.2.3. Apoptosis and Cell-Cycle Analysis

**Figure 8:**
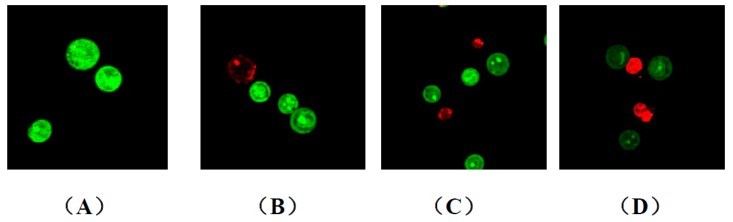
Morphological observation of 2i with AO/EB double staining under fluorescence microscope (630×) on HL-60 cells. (**A**) Cells not treated with **2i** (control); (**B**) cells treated with compound **2i** at 6.25 μM; (**C**) cells treated with compound **2i** at 12.5 μM; (**D**) cells treated with compound **2i** at 25 μM.

Cell-cycle arrest and apoptosis are widely accepted as the main mechanisms of inhibition in tumor formation [19,20]. As HL-60 cells were the most sensitive to all tested compounds, they were selected for testing the most active compound **2i** (at 6.25-, 12.5-, and 25-μM doses), for their effects on apoptosis and cell cycle profile after 48 h. The induction of apoptosis by **2i** was evident from the nuclear morphologic alterations. These were observed by confocal microscopy after labeling the cells with acridine orange (AO)/ethidium bromide (EB). Representative images from four independent experiments are shown in Figure 8. Compared to the control group, chromatin condensation, nuclear fragmentation, and apoptotic bodies were observed in treated cells at concentrations of 6.25, 12.5, and 25 μM. At the highest concentration of 25 μM, the necrosis cell volume was increased, showing uneven orange-red fluorescence and an unapparent outline.

**Figure 9:**
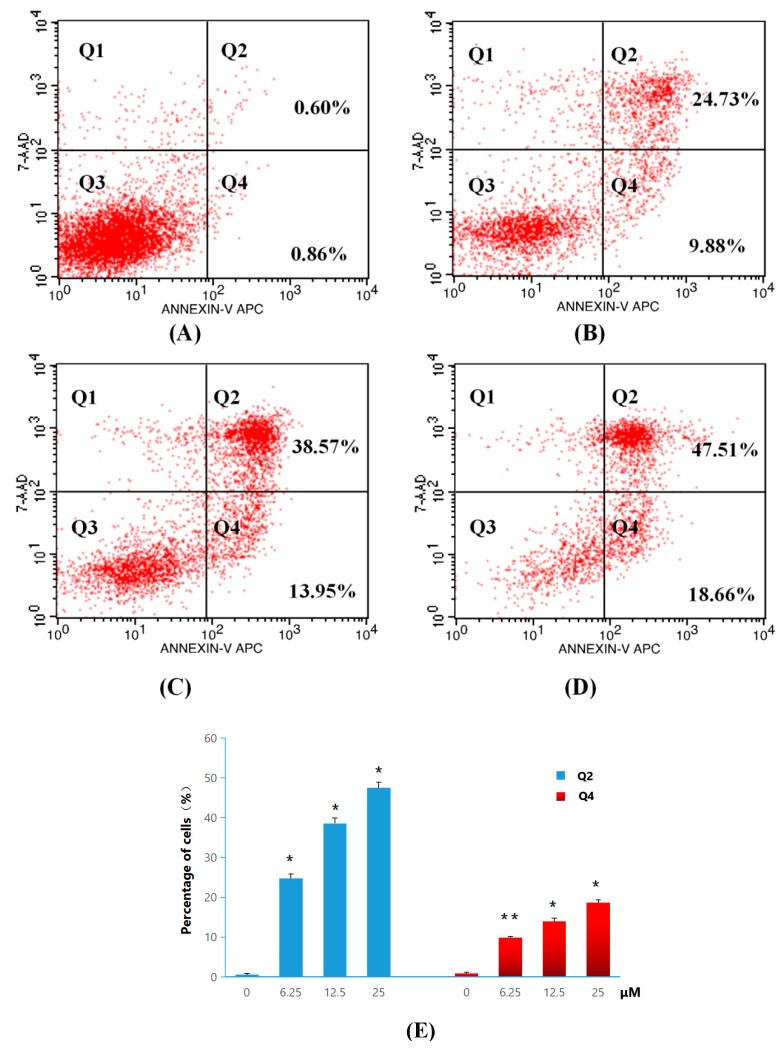
Apoptosis ratio detection of compound 2i for 48 h by Annexin V/PI assay. (**A**) Not treated with compound **2i** (control); (**B**) treated with compound **2i** at 6.25 μM; (**C**) treated with compound **2i** at 12.50 μM for and (**D**) treated with compound **2i** at 25 μM (**E**) The percentage of quadrants Q2 and Q4 are shown as mean ± SD. Data shown in the figure represent three independent experiments (* *p* < 0.05, ** *p* < 0.01).

The extent of apoptosis by flow cytometric analysis of the HL-60 cells labeled with Annexin V-APC/7-AAD was further quantified. For this study, treatment with **2i** at doses of 6.25, 12.5, and 25 μM for 48 h was carried out. Such treatments resulted in 34.61%, 52.52%, and 66.17% apoptotic cells, respectively (Figure 9). The apoptosis ratio for control was found to be 1.46%. The obtained results thus indicate that compound **2i** suppressed cell proliferation by inducing apoptosis, and a considerable dose dependence was observed at concentrations of 6.25, 12.5, and 25 μM. 

**Figure 10:**
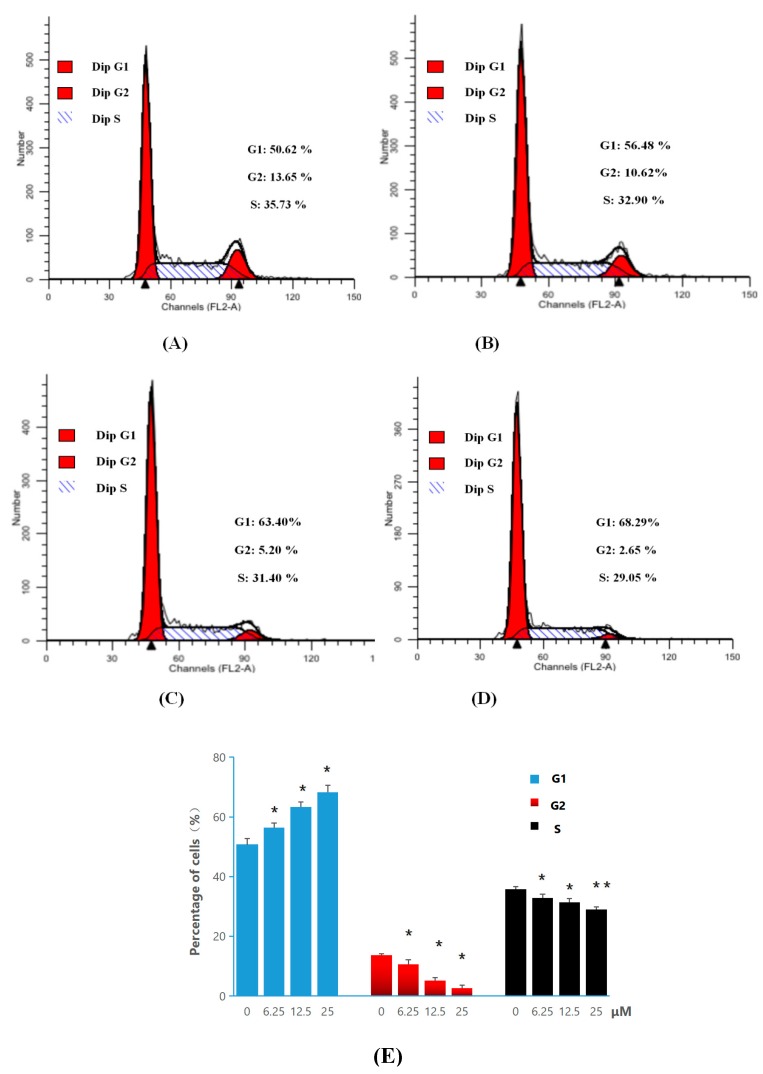
Cell-cycle analysis of HL-60 cells treated with compound **2i** for 48 h, by flow cytometry. (**A**) Not treated with compound **2i** (control); (**B**) treated with compound **2i** at 6.25 μM; (**C**) treated with compound **2i** at 12.50 μM; (**D**) treated with compound **2i** at 25 μM; (**E**) The percentages of each population are shown as mean ± SD. Data shown in the figure are representative of three independent experiments (* *p* < 0.05, ** *p* < 0.01).

Since the induction of apoptosis may be mediated through the regulation of cell cycle, we also examined the effect of **2i** on cell cycle perturbations labeled with propidium iodide (PI). Compared to control, **2i** treatment resulted in an appreciable arrest of HL-60 cells in the G1 phase of the cell cycle, after 48 h of the treatment (Figure 10). The treatment caused an arrest of 56.48% cells in G1 phase of the cell cycle at a 6.25 µM dose, which further increased to 63.40%and 68.29% at higher doses of 12.5 and 25 µM. However, this increase in G1 cell population was accompanied by a decrease of cell number in S and G2 phases. These results indicated that compound **2i** induced a significant cell cycle arrest in G1 phase in a concentration-dependent manner.

## 3. Materials and Methods 

All chemicals used in the study were reagent grade and are commercially available. All yields refer to the yields of isolated products after purification. NMR spectra were measured on a Bruker DRX-500 (^1^H: 500 MHz, ^13^C: 126 MHz) using CDCl_3_ as solvent. Chemical shifts (d) are expressed in parts per million (ppm), and *J* values are given in hertz (Hz). The mass spectra (MS) were obtained on an Agilent 6210 ESI/TOF MS instrument. Melting points were determined using an X-4 apparatus, and were uncorrected. Samples of compounds 2a–2i are available from the authors.

### 3.1. Synthesis Methods

#### General Procedure for the Synthesis of 4-Acyloxy Derivatives of Robustic Acid **2a**–**2j**

Robustic acid (0.19 g, 0.5 mmol) was dissolved in CH_3_CN (10 mL), followed by the addition of acyl chloride derivative (1 mmol) and pyridine (0.161 μL). After 15–210 min refluxing with stirring (monitored by thin-layer chromatography), the reaction mixture was cooled to room temperature. The solvent was removed and recrystallization with ethanol was carried out. The formed crystals (**2a**–**2j**) were filtered and dried under vacuum. 

4-*O*-Benzoyl-robustic acid (**2a**): reaction time 2.5 h, white powder, isolated yield 67%, m.p. 266.4–273.4 °C. ^1^H NMR (500 MHz, CDCl_3_) δ 8.09–8.02 (m, 2H, ArH), 7.64 (t, *J* = 7.4 Hz, 1H, ArH), 7.50 (q, *J* = 7.9, 7.3 Hz, 2H, ArH), 7.42 (d, *J* = 8.8 Hz, 1H, ArH), 6.89 (d, *J* = 8.8 Hz, 2H, ArH), 6.70 (s, 1H, ArH), 6.49 (d, *J* = 10.1 Hz, 1H, pyran ring), 5.70 (d, *J* = 10.1 Hz, 1H, pyran ring), 3.79 (s, 3H, -OCH_3_), 3.56 (s, 3H, -OCH_3_), 1.49 (s, 6H, 2-CH_3_); ^13^C NMR (126 MHz, CDCl_3_) δ 165.53, 163.48, 161.83, 159.52, 157.56, 154.58, 154.30, 133.79, 131.19, 130.77, 130.72, 130.14, 130.12, 128.66, 128.62, 123.75, 122.55, 117.12, 115.88, 115.83, 113.71, 110.48, 108.18, 101.28, 101.12, 63.55, 55.21, 28.21, 28.16. HR-MS (ESI) *m/z*: calculated for C_29_H_24_O_7_ [M + H]^+^: 485.1595, found: 485.1599.

4-*O*-Trichloroacetyl-robustic acid (**2b**): reaction time 2.0 h, white powder, isolated yield 41%, m.p. 218.2–223.1 °C. ^1^H NMR (500 MHz, CDCl_3_) δ 7.34 (d, *J* = 8.3 Hz, 1H, ArH), 7.29 (s, 1H, ArH), 6.97 (d, *J* = 8.3 Hz, 2H, ArH), 6.70 (s, 1H, ArH), 6.58 (d, *J* = 10.0 Hz, 1H, pyran ring), 5.77 (d, *J* = 10.1 Hz, 1H, pyran ring), 3.87 (s, 3H, -OCH_3_), 3.84 (s, 2H, -OCH_3_), 1.51 (s, 6H, 2-CH_3_); ^13^C NMR (126 MHz, CDCl_3_) δ 161.23, 160.10, 158.72, 158.15, 154.13, 153.71, 152.56, 131.39, 131.26, 130.99, 124.02, 121.15, 115.84, 114.02, 113.95, 112.63, 110.54, 108.50, 102.66, 101.31, 64.42, 55.35, 28.20, 28.00. HR-MS (ESI) *m/z*: calculated for C_24_H_19_C_l3_O_7_ [M + H]^+^: 525.0269, found: 525.0267.

4-*O*-(*p*-Ethoxy benzoyl)-robustic acid (**2c**): reaction time 2.0 h, white crystal, isolated yield 56%; m.p. 226.3–228.7 °C. ^1^H NMR (500 MHz, CDCl_3_) δ 7.99 (d, *J* = 8.9 Hz, 2H, ArH), 7.41 (d, *J* = 8.8 Hz, 2H, ArH), 6.93 (d, *J* = 8.9 Hz, 2H, ArH), 6.87 (d, *J* = 8.8 Hz, 2H, ArH), 6.71–6.64 (m, 1H, ArH), 6.49 (d, *J* = 9.8 Hz, 1H, pyran ring), 5.69 (d, *J* = 10.1 Hz, 1H, pyran ring), 4.11 (q, *J* = 7.0 Hz, 2H, -OCH_2_), 3.76 (s, 3H, -OCH_3_), 3.58 (s, 3H, -OCH_3_), 1.47 (s, 6H, 2-CH_3_), 1.47 – 1.41 (m, 3H, -CH_3_); ^13^C NMR (126 MHz, CDCl_3_) δ 163.56, 163.24, 161.90, 159.46, 157.50, 154.87, 154.27, 152.38, 132.35, 132.32, 131.21, 130.69, 123.77, 122.69, 120.49, 117.05, 115.92, 114.39, 114.38, 113.64, 112.71, 110.50, 108.12, 104.20, 101.15, 63.88, 63.61, 55.17, 28.18, 28.13, 14.65. HR-MS (ESI) *m/z*: calculated for C_31_H_28_O_8_ [M + H]^+^: 529.1857, found: 529.1861.

4-*O*-(*p*-Trifluoromethyl benzoyl)robustic acid (**2d**): reaction time 1.0 h, white crystal, isolated yield 81%; m.p. 225.6–228.5 °C. ^1^H NMR (500 MHz, CDCl_3_) δ ^1^H NMR (500 MHz, CDCl_3_) δ 8.16 (d, *J* = 7.9 Hz, 2H, ArH), 7.77 (d, *J* = 8.1 Hz, 2H, ArH), 7.40 (d, *J* = 8.8 Hz, 2H, ArH), 6.90 (d, *J* = 8.9 Hz, 2H, ArH), 6.71 (s, 1H, ArH), 6.48 (d, *J* = 10.1Hz, 1H, pyran ring), 5.72 (d, *J* = 10.1 Hz, 1H, pyran ring), 3.79 (s, 3H, -OCH_3_), 3.55 (s, 3H, -OCH_3_), 1.50 (s, 6H, -CH_3_); ^13^C NMR (126 MHz, CDCl_3_) δ 162.30, 161.62, 159.66, 157.88, 157.72, 154.53, 154.27, 154.09, 152.03, 131.11, 130.99, 130.95, 130.47, 130.44, 130.44, 125.79, 125.75, 125.72, 125.69, 123.64, 122.30, 117.18, 115.70, 115.66, 113.78, 112.73, 112.69, 110.37, 108.24, 103.50, 101.38, 101.21, 63.53, 55.22, 28.24, 28.14. HR-MS (ESI) *m/z*: calculated for C_30_H_23_F_3_O_7_ [M + H]^+^: 553.1469, found: 553.1469.

4-*O*-Thenoyl robustic acid (**2e**): reaction time 2.0 h, brown powder, isolated yield 46%; m.p. 171.5–182.3 °C. ^1^H NMR (500 MHz, CDCl_3_) δ 7.37–7.20 (m, 3H, ArH), 6.99–6.93 (m, 1H, ArH), 6.89 (d, *J* = 8.3 Hz, 2H, ArH), 6.83 (s, 1H, ArH), 6.67 (s, 1H, ArH), 6.57 (d, *J* = 10.1 Hz, 1H, pyran ring), 5.75 (d, *J* = 10.1 Hz, 1H, pyran ring), 3.94 (s, 3H, -OCH_3_), 3.86 (s, 3H, -OCH_3_), 3.78 (s, 3H, -OCH_3_), 1.50 (s, 6H, -CH_3_); ^13^C NMR (126 MHz, CDCl_3_) δ 167.02, 161.66, 159.62, 157.63, 154.25, 154.22, 152.18, 131.05, 130.94, 127.52, 126.95, 125.38, 123.62, 122.30, 117.07, 115.76, 113.80, 112.64, 110.37, 108.28, 103.62, 101.33, 63.59, 55.29, 34.71, 28.20, 28.19. HR-MS (ESI) *m/z*: calculated for C_27_H_22_O_7_S [M+Na]^+^: 513.0978, found: 513.0985.

4-*O*-(*p*-Fluorobenzoyl)robustic acid (**2f**): reaction time 50 min, white crystal, isolated yield 63%; m.p. 236.8–239.6 °C. ^1^H NMR (500 MHz, CDCl_3_) δ 8.14–7.99 (m, 2H, ArH), 7.40 (d, *J* = 8.8 Hz, 2H, ArH), 7.16 (t, *J* = 8.6 Hz, 2H, ArH), 6.89 (d, *J* = 8.8 Hz, 2H, ArH), 6.70 (s, 1H, ArH), 6.49 (d, *J* = 10.0 Hz, 1H, pyran ring), 5.71 (d, *J* = 10.1 Hz, 1H, pyran ring-H), 3.78 (s, 3H, -OCH_3_), 3.57 (s, 3H, -OCH_3_), 1.49 (s, 6H, -CH_3_); ^13^C NMR (126 MHz, CDCl_3_) δ 167.26, 165.22, 162.51, 161.74, 159.57, 157.63, 154.41, 154.27, 152.18, 132.79, 132.72, 131.15, 130.85, 124.78, 123.70, 122.48, 117.13, 116.05, 115.87, 115.79, 113.71, 112.69, 110.42, 108.18, 103.82, 101.28, 63.55, 55.20, 28.21, 28.14. HR-MS (ESI) *m/z*: calculated for C_29_H_23_FO_7_ [M + H]^+^: 503.1501, found: 503.1504. 

4-*O*-(*p*-Nitrobenzoyl)robustic acid (**2g**): reaction time 15 min, yellow powder, isolated yield 85%; m.p. 239.5–241.9 °C. ^1^H NMR (500 MHz, CDCl_3_) δ 8.35 (d, *J* = 8.8 Hz, 2H, ArH), 8.22 (d, *J* = 8.8 Hz, 2H, ArH), 7.39 (d, *J* = 8.8 Hz, 2H, ArH), 6.90 (d, *J* = 8.8 Hz, 2H, ArH), 6.72 (s, 1H, ArH), 6.47 (d, *J* = 10.2 Hz, 1H, pyran ring), 5.73 (d, *J* = 10.2 Hz, 1H, pyran ring), 3.79 (s, 3H, -OCH_3_), 3.55 (s, 3H, -OCH_3_), 1.50 (s, 6H, -CH_3_); ^13^C NMR (125 MHz, CDCl_3_) δ 167.26, 165.22, 162.51, 161.74, 159.57, 157.63, 154.41, 154.27, 152.18, 132.79, 131.15, 130.85, 124.78, 123.70, 122.48, 117.13, 116.05, 115.87, 115.79, 113.71, 112.69, 110.42, 108.18, 103.82, 101.28, 63.55, 55.20, 28.21, 28.14. HR-MS (ESI) *m/z*: calculated for C_29_H_23_NO_7_ [M + H]^+^: 530.1446, found: 530.1451.

4-*O*-(*p*-Ethylbenzoyl)robustic acid (**2h**): reaction time 1.5 h, white powder, isolated yield 65%; m.p. 246.9–250.2 °C. ^1^H NMR (500 MHz, CDCl_3_) δ 7.97 (d, *J* = 7.9 Hz, 2H), 7.41 (d, *J* = 8.4 Hz, 2H, ArH), 7.31 (d, *J* = 7.9 Hz, 2H, ArH), 6.89 (d, *J* = 8.4 Hz, 2H, ArH), 6.70 (s, 1H, ArH), 6.49 (d, *J* = 10.1 Hz, 1H, pyran ring), 5.69 (d, *J* = 10.1 Hz, 1H, pyran ring), 3.79 (s, 3H, -OCH_3_), 3.57 (s, 3H, -OCH_3_), 2.75 (q, *J* = 7.6 Hz, 2H, -CH_2_), 1.49 (s, 6H, -CH_3_), 1.30 (t, *J* = 7.6 Hz, 3H, -CH_3_); ^13^C NMR (125MHz, CDCl_3_) δ ^13^C NMR (126 MHz, CDCl_3_) δ 163.53, 161.89, 159.47, 157.51, 154.72, 154.29, 152.31, 150.84, 131.21, 130.67, 130.30, 128.15, 125.97, 122.64, 117.10, 115.91, 115.87, 113.69, 112.67, 104.05, 101.24, 63.60, 55.20, 29.02, 28.18, 15.01. HR-MS (ESI) *m/z*: calculated for C_31_H_28_O_7_ [M + H]^+^: 513.1908, found: 513.1906.

4-*O*-(4-Bromo-2-fluorobenzoyl) robustic acid (**2i**): reaction time 30 min, white powder, isolated yield 71%; m.p. 250.8–251.9 °C. ^1^H NMR (500 MHz, CDCl_3_) δ 7.82 (t, *J* = 8.1 Hz, 1H, ArH), 7.51–7.33 (m, 4H, ArH), 6.91 (d, *J* = 8.5 Hz, 2H, ArH), 6.70 (s, 1H, ArH), 6.50 (d, *J* = 10.1 Hz, 1H, pyran ring), 5.72 (d, *J* = 10.1 Hz, 1H, pyran ring), 3.81 (s, 3H, -OCH_3_), 3.61 (s, 3H, -OCH_3_), 1.50 (s, 6H, -CH_3_); ^13^C NMR (125 MHz, CDCl_3_) δ 162.79, 161.68, 160.67, 160.13, 160.09, 159.63, 157.66, 154.24, 153.79, 152.11, 133.64, 131.28, 130.86, 127.94, 127.91, 123.86, 122.27, 121.09, 120.88, 117.13, 115.77, 113.69, 112.68, 110.51, 108.17, 103.56, 101.31, 101.18, 63.44, 55.23, 28.26, 28.13. HR-MS (ESI) *m/z*: calculated for C_29_H_22_BrFO_7_ [M+Na]^+^: 603.0425, found: 603.0429.

4-*O*-Acetylrobustic acid (**2j**): reaction time 3.5 h, white crystal, isolated yield 51%; m.p. 189.9–192.1 °C (lit. [24], m.p. 198–199 °C; lit. [25], m.p. 194–196 °C). ^1^H NMR (600 MHz, CDCl_3_) δ 7.35 (d, *J* = 8.7 Hz, 2H, ArH), 7.02–6.92 (m, 2H, ArH), 6.88 (d, *J* = 7.9 Hz, 1H, ArH), 6.67 (s, 1H, ArH), 6.57 (d, *J* = 7.8 Hz, 1H, ArH), 6.02 (s, 1H, pyran ring), 5.74 (d, *J* = 10.1 Hz, 1H, pyran ring), 3.86 (s, 3H, -OCH_3_), 3.82 (s, 3H, -OCH_3_), 2.17 (s, 3H, -COCH_3_), 1.51 (s, 6H, -CH_3_); ^13^C NMR (150 MHz, CDCl_3_)δ 167.54, 161.74, 159.63, 157.55, 154.28, 154.21, 152.08, 131.11, 130.96, 123.64, 122.63, 116.94, 115.73, 115.69, 113.77, 112.65, 110.39, 108.24, 103.74, 101.35, 101.22, 63.52, 55.28, 28.19, 28.00, 20.46. HR-MS (ESI) m/z: calculated for C_24_H_22_O_7_ [M+Na]^+^: 445.1258, found: 445.1256.

### 3.2. Biological Activity

#### 3.2.1. Antiproliferative Activity

The MTS (3-(4,5-dimethylthiazole-2-yl)-5-(3-carboxymethoxyphenyl)-2-(4-sulfophenyl)-2H- tetrazolium) (Promega, Madison, WI, USA) assay was used to determine the IC_50_ value for all compounds, according to [26]. We used a panel of five human carcinoma cell lines and two normal cells including HL-60, A549, SMMC-7721, HepG2, Hela, LO2 and BEAS-2B. Cells were seeded in 96-well plates at a density of 3 × 10^3^–1.5 × 10^4^ cells/well in 100 μL medium (DMEM or RMPI1640) and incubated for 12–24 h at 37 °C. Subsequently, cells were exposed to drugs in an additional 100 μL medium and were further incubated at 37 °C for 48 h. In brief, 100 μL MTS reagent supplement with 100 μL medium was added directly to the wells and cells were incubated at 37 °C for a minimum of 2 h. Colorimetric measurements were recorded at 492 nm. Background absorbance was corrected using triplicate sets of wells, and cells were incubated at 37 °C for a minimum of 2 h. Colorimetric measurements were recorded at 492 nm. Background absorbance was corrected using triplicate sets of wells containing medium only (no cells), and MTS reagent was added per experimental well. Each experiment was performed at least three times, and values in results are represented as mean ± SD. 

#### 3.2.2. Topo I Inhibitory Activity

Supercoiled pBR32 DNA was used as a substrate to determine the Topo I catalytic activity. Commercial samples of Topo I and pBR322 were obtained from Takara Bio Inc. The buffer solutions were prepared using 500 mM KAc, 200 mM Tris-Ac, 100 mM Mg(Ac)_2_, and 1 mg/mL BSA. The procedure used for determination of enzyme inhibitory activity was similar to one reported by Xu et al. [27].

#### 3.2.3. General Procedure for AO/EB Staining

Apoptosis was determined by nuclear morphology. Cells were fixed and stained with AO/EB according to the manufacturer’s instruction (KGA501, KeyGEN BioTECH, Nanjing, China). HL-60 cells were seeded in six-well plates with sterile cover slips. The concentration of cells used per well was 5 × 10^5^–6 × 10^6^ cells/2 mL. To facilitate growth, the medium was replaced with fresh medium (RPMI1640) plus 10% fetal bovine serum. The fresh medium was supplemented with compound **2i** (6.25, 12.50, and 25 μM). Post treatment, 25 μL of cell suspension was collected on a glass slide by inverting the cover slip and stained with 10 μL of AO/EB stain (100 mg/mL). Finally, stained nuclei were observed immediately under a fluorescence microscope (BX41, Nikon, Tokyo, Japan).

#### 3.2.4. General Procedure for Apoptosis Ratio Determination

For determination of apoptosis ratios by application of flow cytometry analysis, the manufacturer’s protocol (KGA1024, KeyGEN BioTECH, Nanjing, China) for Annexin-V APC/7-AAD double-stain assay was used. The HL-60 cells were grown at a density of 5 × 10^5^ cells/mL in six-well plates (Costar), and were treated with **2i** (6.25, 12.50, and 25 μM) for 72 h. The cells were trypsinized and washed with PBS. Subsequently, the prepared HL-60 cells (5 × 10^5^ cells/mL) were collected and resuspended in 500 μL of binding buffer containing 5 μL of Annexin V-APC. The suspension was shaken well, and 5 μL of 7-AAD was added to it. The suspension was then incubated for 5–15 min in the dark at room temperature. The suspensions were then analyzed by flow cytometry (FACS Cali-bur, Becton Dickinson, San Jose, CA, USA).

#### 3.2.5. General Procedure for Cell Cycle Analysis

The intercalating blue-excited dye propidium iodide (PI) was used in this study. HL-60 cell cultures were seeded within six-well plates (Costar). Then, the cells were treated with **2i** (6.25, 12.50, and 25 μM) in DMEM complete medium for 72 h. After the incubation time elapsed, the harvest procedure was performed as follows: the supernatant medium was transferred to centrifuge tubes. To the medium, 0.25% trypsin (Sigma) was added, until all cells were detached. The cell suspension was then washed twice with PBS and resuspended. About 1 mL of 70% ethanol was quickly added to the cell suspension (5 × 10^5^ cells/mL), which was then incubated at 4 °C overnight. The samples were treated with 100 μL RNase A at 37 °C for 30 min and then stained with 400 μL PI solution. After another 30 min of incubation at 4 °C, the resulting nuclei suspension was analyzed with a flow cytometer (FACS Verse, Becton Dickinson, San Jose, CA, USA) at 488 nm excitation for red fluorescence of PI.

### 3.3. Molecular Docking

Surflex-Dock in Sybyl 2.0 was used for molecular docking. This system performs molecular docking functions aided by the generation of an idealized active site (Protocol), consisting of dummy atoms that guide the docking process. The crystal structures of DNA-Topo I complex was downloaded from the RCSB website (https://www.rcsb.org/structure/1T8I) (PDB ID: 1T8I). The proteins were then imported into the Surflex-Dock and prepared for docking using the biopolymer preparation tool, according to the following criteria: H-Addition, H-Bond, removal of water molecules, termini treatment, charge, protonation. 

The structures of the compounds were sketched using ChemBioOffice version 14.0 by PerkinElmer (Waltham, MA, USA). The alignments of the training set molecules were derived using FlexS in SYBYL. All values were filled with valence, and Gasteiger–Marsili charges were calculated for each compound. Ultimately, ligand docking under the Surflex-Dock GeomX precision was performed to generate a grid of proteins and ligands. The docking results were then imported into LigPlot^+^ 2.1, and the combinations between compounds and proteins were discussed in terms of H-Bond, conjugate action, and hydrophilic or hydrophobic action.

## 4. Conclusions

A new series of linear pyranocoumarin derivatives were synthesized, and their anti-cancer activities were evaluated. The biological screening results indicated that most of the synthesized compounds exhibited potent in vitro anti-cancer activity, which were better than original nucleus robustic acid. Among them, compounds **2d**, **2g**, and **2i** were showed excellent anti-cancer activity with respective IC_50_ values of 21.04 ± 0.43, 16.63 ± 0.12, and 16.38 ± 0.27 μM against HL-60 cells. These values were estimated in comparison to the positive control cisplatin (14.23 ± 0.56 μM) and CPT (12.25 ± 1.06 μM). And these compounds were much less toxic to non-cancerous cells when comparing with the two positive controls. In addition to this, docking studies of **2d**, **2g**, and **2i** were performed, and they all exhibited good binding affinities with relatively high total scores of 8.76, 10.02, and 8.89. The inhibitory activities of the compounds against Topo I indicated that **2g** and **2i** were showed significant inhibitory activity, even at the low concentration of 0.1 mM. Compound **2d** aslo exhibited moderate inhibitory activity. These results indicate that the anti-tumor activity of these three compounds may be related to Topo I inhibitory activity. However, the results of the docking assay of other compounds do not exactly correlate with the Topo I inhibitory and anti-tumor activity. This may be the result of other pharmacological mechanisms. Later, compound **2i** was selected as the representative compound for further investigation, due to its properties of suppressing cell proliferation by inducing apoptosis and arresting HL-60 cells in the G1 stage. These findings may contribute to the identification of novel linear pyranocoumarins that can serve as the new lead compounds for anti-tumor drugs.

## Figures and Tables

**Table 1:** IC_50_ values of the synthesized compounds and controls obtained for anti-proliferative activity on different tumor cell lines (μM).

Compounds	HL-60	A-549	SMMC-7721	HepG2	Hela	LO2	BEAS-2B
**2a**	>100	98.42 ± 4.18	40.24 ± 1.38	>100	>100	>100	>100
**2b**	>100	>100	>100	>100	>100	>100	>100
**2c**	>100	>100	>100	>100	>100	>100	>100
**2d**	21.04 ± 0.43	53.33 ± 1.37	36.79 ± 1.06	40.12 ± 1.36	37.03 ± 0.97	>100	>100
**2e**	80.19 ± 3.72	91.63 ± 2.21	68.18 ± 3.92	58.99 ± 3.11	>100	>100	>100
**2f**	>100	>100	66.18 ± 5.70	87.27 ± 2.14	65.07 ± 1.27	>100	>100
**2g**	16.63 ± 0.12	40.26 ± 0.62	30.47 ± 1.59	55.87± 0.42	27.45 ± 0.38	20.79 ± 0.97	>100
**2h**	>100	>100	92.01 ± 6.10	>100	>100	>100	>100
**2i**	16.38 ± 0.27	37.06 ± 0.61	31.58 ± 1.25	44.23 ± 0.89	67.45 ± 1.24	>100	>100
**2j**	87.44 ± 7.12	>100	78.15 ± 4.35	>100	>100	>100	>100
RA	>100	>100	>100	>100	>100	>100	>100
CPT	12.25 ± 1.06	15.34 ± 1.22	18.9 ± 4.12	1.3 ± 0.62	0.5 ± 0.08	0.030 ± 0.002	2.159 ± 0.47
cisplatin	14.23 ± 0.56	25.40 ± 1.06	1.32 ± 0.97	10.59 ± 1.40	5.83 ± 0.14	<0.008	57.66 ± 3.10

**Table 2:** Interactions between DNA Topo I (from PDB ID 1T8I) and target compounds.

Compound	Amino Acid	Type	Hydrophobic Residues	Total Score ^a^
**2a**	DA113	π–π	LYS374, GLU356, DC112, TGP11, DT10, TYR426, ALA351, ASN352, MET428, LYS425	8.4481
	DC112	π–π		
	DA113	Hydrogen Bond		
**2b**	TGP11	π–π	ASP533, THR718, TGP11, DT10, GLU356, ASN352, DC112, DG12	6.9191
	DA113	π–π		
	ARG364	Hydrogen Bond		
	DA113	Hydrogen Bond		
**2c**	TGP11	π–π	ASP533, TGP11, GLU356, DC112, LYS532, HIS632, DT10, ASN722, ASN352, DA113	8.9089
	DA113	π–π		
	DC112	π–π		
	DT10	π–π		
	THR718	Hydrogen Bond		
	ARG364	Hydrogen Bond		
**2d**	TGP11	π–π	ASN722, DT10, TGP11, ALN351, ASN352, GLU356, DA113, ASP533, PTR723, ARG488, LYS532, HIS632	8.7600
	DA113	π–π		
	DT10	π–π		
	ARG364	Hydrogen Bond		
	THR718	Hydrogen Bond		
**2e**	TGP11	π–π	ASN722, DT10, TGP11, ALN351, ASN352, GLU356, DA113, ASP533, PTR723, ARG488, LYS532, HIS632	8.6166
	DA113	π–π		
	DC112	π–π		
	ARG364	Hydrogen Bond		
	DA113	Hydrogen Bond		
**2f**	TGP11	π–π	DC112, TGP11, THR718, DT10, TRP416, GLU356, LYS354, ASN352	8.5897
	DA113	π–π		
	DT10	π–π		
	DA113	Hydrogen Bond		
	ARP364	Hydrogen Bond		
**2g**	DC112	π–π	ILE427, TGP11, DT10, DA113, ARG364, DC112, PRO357, GLU356, LYS374, GLU418, TYR426	10.0229
	TGP11	π–π		
	DA113	π–π		
	MET428	Hydrogen Bond		
	LYS425	Hydrogen Bond		
**2h**	DT10	π–π	DC112, TGP11, THR718, DT10, LYS436, PRO431, ASN352, TRP416, LYS354, GLU356	9.2983
	TGP11	π–π		
	DA113	π–π		
	DA113	Hydrogen Bond		
	ARG364	Hydrogen Bond		
**2i**	DA113	π–π	DT10, TGP11, THR718, DC112, LYS354, GLU356, TRP416, ASN352	8.8944
	TGP11	π–π		
	DT10	π–π		
	DA113	Hydrogen Bond		
	ARG364	Hydrogen Bond		
**2j**	TGP11	π–π	DC112, TYR426, LYS425, ASN352, THR718, DT10, TGP11	8.4305
	DA113	π–π		
	ARG364	Hydrogen Bond		
	DA113	Hydrogen Bond		
RA	TGP11	π–π	ARG364, ASP533, TGP11, DA113, ALA351, ASN352, LEU429, MET428	7.8183
	DA113	π–π		
	DT10	Hydrogen Bond		
CPT	TGP11	π–π	DC112, DA113, TGP11, DT10, DG12	10.3269
	DA113	π–π		
	DC112	π–π		
	ASP533	Hydrogen Bond		
	TGP11	π–π		

^a^ Total score represents the binding affinity in units of -logK_d_.
